# Comparison and Optimization of Quantification Methods for *Shigella flexneri* Serotype 6 O-antigen Containing Galacturonic Acid and Methyl-Pentose

**DOI:** 10.3390/ijms222212160

**Published:** 2021-11-10

**Authors:** Maria Michelina Raso, Oscar Vassallo, Francesca Micoli, Carlo Giannelli

**Affiliations:** 1 GSK Vaccines Institute for Global Health (GVGH) S.r.l., Via Fiorentina 1, 53100 Siena, Italy; maria-michelina.m.raso@gsk.com (M.M.R.); oscar.vassallo@merckgroup.com (O.V.); francesca.x.micoli@gsk.com (F.M.); 2 Merck, Via Einaudi 11, 00012 Guidonia, Italy

**Keywords:** *S. flexneri* O-antigen, HPAEC-PAD, Dische colorimetric method, quantification, polysaccharide, vaccine, design of experiment, galacturonic acid, methyl-pentose

## Abstract

*Shigella* is a leading diarrheal cause of morbidity and mortality worldwide, especially in low- and middle-income countries and in children under five years of age. Increasing levels of antimicrobial resistance make vaccine development an even higher global health priority. *S. flexneri* serotype 6 is one of the targets of many multicomponent vaccines in development to ensure broad protection against *Shigella*. The O-antigen (OAg) is a key active ingredient and its content is a critical quality attribute for vaccine release in order to monitor their stability and to ensure appropriate immune response. Here, the optimization of two methods to quantify *S. flexneri* 6 OAg is reported together with the characterization of their performances. The optimized Dische colorimetric method allows a tenfold increment of the sensitivity with respect to the original method and is useful for fast analysis detecting selectively methyl-pentoses, as rhamnose in *S. flexneri* 6 OAg. Also, a more specific HPAEC-PAD method was developed, detecting the dimer galacturonic acid-galactosamine (GalA-GalN) coming from *S. flexneri* 6 OAg acid hydrolysis. These methods will facilitate characterization of *S. flexneri* 6 OAg based vaccines. The colorimetric method can be used for quantification of other polysaccharide containing methyl-pentoses, and the HPAEC-PAD could be extended to other polysaccharides containing uronic acids.

## 1. Introduction

*Shigella* infection is a leading bacterial cause of moderate to severe diarrhea (MSD) throughout the world. The bacteria are facultative intracellular pathogens with high specificity for the human host in which they cause Shigellosis, commonly known as bacillary dysentery, which is characterized by watery diarrhea, fever, abdominal pain, and bloody and mucusy stools [1]. There are approximately 270 million cases with 212,438 total deaths per year, most in low-and middle-income countries. Some 64,000 of these cases are children younger than five years (LMIC) [2]. There are four different *Shigella* species: *S. boydii*, *S. dysenteriae*, *S. flexneri*, and *S. sonnei*. The first three species are typed into more than 50 different serotypes or sub-serotypes based on antigenic variation of the O-antigen (OAg) component of their lipopolysaccharides (LPS) [3]. Incident data from specific sites of the Global Enteric Multicenter Study (GEMS) in sub-Saharan Africa and South Asia reported 24% cases caused by *S. sonnei* and 66% by *S. flexneri*, mostly by serotypes 1b, 2a, 3a, and 6 [4]. Increasing levels of multidrug-resistance [5,6] limit the effectiveness of antibiotics, making this disease an even higher priority for vaccine development [7,8]. Currently, no vaccines are widely available against *Shigella*, but several candidates are at different stages of development, including subunit vaccines and killed or live-attenuated bacteria [9,10]. Studies in animal models and humans have demonstrated that protection by immunization is feasible. Serum and mucosal antibody responses to *Shigella* are predominantly directed against the serotype-specific *Shigella* OAg, and many *Shigella* vaccine candidates target the OAg [9,11]. In particular, research is ongoing to develop multicomponent vaccines with broad protection against different *Shigella* serotypes. Many of them target *S. flexneri* serotype 6 among the *S. flexneri* components further than *S. sonnei* [12].

The manufacture of vaccines requires good characterization and quality control of all its components. OAg content is one of the critical quality attributes of an OAg-based vaccine and a method for *S. flexneri* 6 OAg quantification is fundamental for vaccine release in order to monitor its stability and ensure an appropriate immune response.

**Figure 1:**
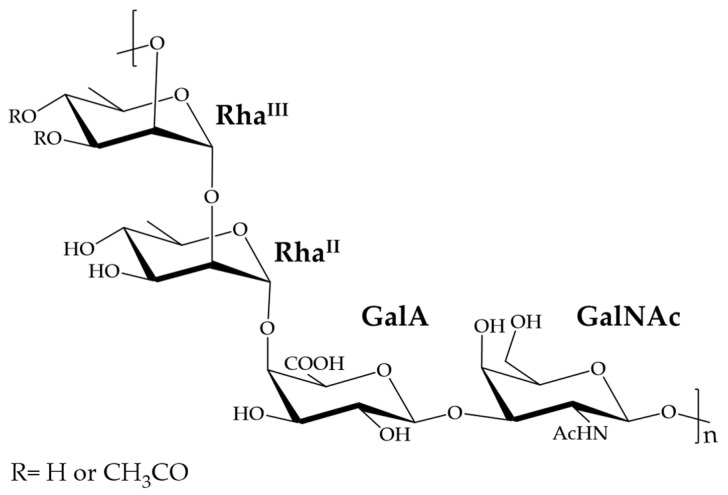
*S. flexneri* serotype 6 OAg repeating unit (RU) structure.

*S. flexneri* 6 OAg is constituted by a linear polysaccharide backbone [2)-α-L-Rha*p*^III^-(1→2)-α-L-Rha*p*^II^-(1→4)-β-D-Gal*p*A-(1→3)-β-D-Gal*p*NAc-(1→]_n_, with Rha^III^ variably O-acetylated in position 3 or 4 (Figure 1) [13].

Colorimetric methods such as Dische [14,15] or anthrone test [16], commonly used for carbohydrates quantitative analysis, have been proposed for *S. flexneri* 6 OAg quantification [17,18]. In this study, the Dische colorimetric method was further improved and implemented for *S. flexneri* 6 OAg quantification.

Another common and more specific procedure for sugar quantification in polysaccharide based vaccines consists of acid hydrolysis of the polysaccharide followed by released sugar monomers quantification by HPAEC-PAD [19]. Here, for the first time, such methodology has been extended and optimized for *S. flexneri* 6 OAg quantification. Recently we have developed a novel method based on Trifluoroacetic/hydrochloric acid (TFA/HCl) hydrolysis followed by HPAEC-PAD analysis for quantification of 2-amino uronic acids [20]. As *S. flexneri* 6 OAg contains galacturonic acid (GalA), we started from the idea to apply a similar methodology to it.

The Design of Experiment (DoE) statistical tool has been used to facilitate identification of optimal working conditions of the quantification methods investigated.

The methods developed can be applied to other polysaccharide-based vaccines containing methyl-pentoses (6-deoxy-hexoses) or uronic acids.

## 2. Results

### 2.1. Dische Colorimetric Method

The Dische colorimetric method [14,15] used to quantify methyl-pentoses was reported in pharmacopeia [21] to be used in the range of 2–15 μg/mL of rhamnose (Rha). It is described to add 4.5 mL of a cooled H_2_SO_4_/water 6:1 mixture to 1 mL sample, then the mixture is warmed in a water bath for a few minutes and finally, at room temperature, 0.1 mL of a 190 mM cysteine solution is added. After 2 h at room temperature, the absorbances at two different wavelengths are read to subtract the absorbance coming from hexoses eventually present in the sample [14].

Here, the possibility to add H_2_SO_4_ only to the sample, instead of a 6:1 mixture with water, was evaluated both to simplify the procedure and to increase the sensitivity of the method. After some preliminary tests, a DoE was performed using a response surface central composite design (rotatable, alpha 1.68179), with six replicates of the center point and one replicate of the axial/factorial points for a total of 20 runs executed in randomized order. The H_2_SO_4_/water final ratio, the warming time prior to cysteine addition and the cysteine quantity were the factors evaluated respectively in the range 1.3–2.7; 5–15 min and 18–32 μL of a 1 M solution (Table A1).

The experimental work was conducted in parallel on fucose and glucose samples to understand respectively the effect on color formation (measured as Δ absorbance (ABS), see materials and methods section) for methyl-pentoses and hexoses.

For glucose, to elaborate the data, a response surface with a quadratic model was chosen and the data were square root transformed before analysis. Not significant terms (*p*-value > 0.05) were removed from the model using a backward elimination process (statistical analysis and results are reported in Figure A1).

**Figure 2:**
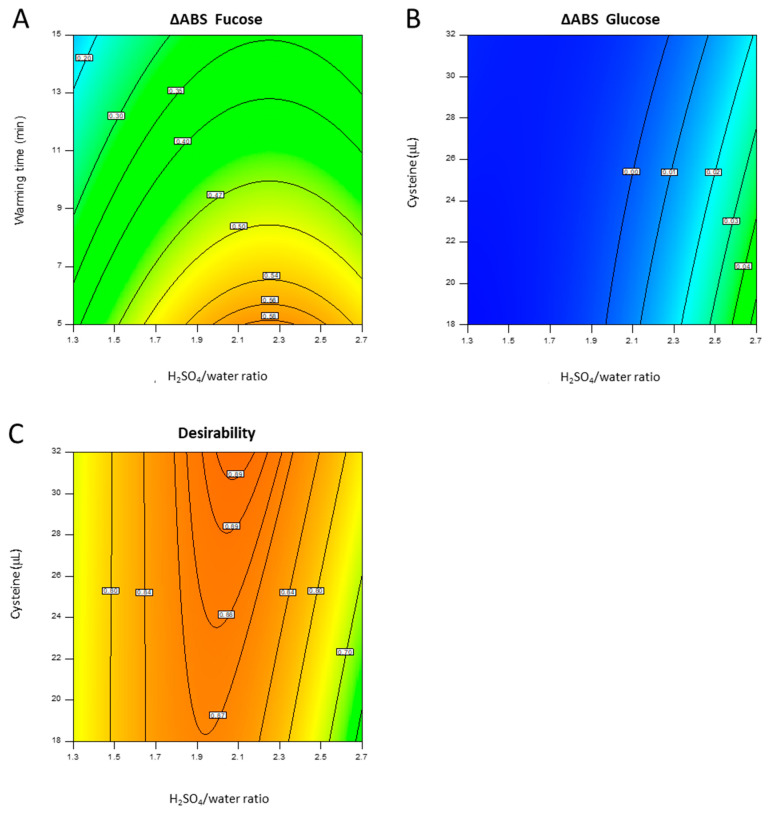
2D contour plots for fucose response (**A**), glucose response (**B**) and for desirability obtained for 5 min warming time (**C**).

A quadratic model was obtained with responses not dependent from warming time; the response was quite flat for quite all the design space and increased with low cysteine concentration and high sulfuric acid/water ratio (Figure 2B).

The model for glucose response (adjusted-R^2^ 0.8628; not significant lack of fit *p* = 0.4316) was used in the optimization of the response even if the residuals were not normally distributed (Anderson Darling *p* = 0.025) after applying data transformations.

For fucose, to elaborate the data, a response surface with a quadratic model was chosen, and not significant terms (*p*-value > 0.05) were removed from the model using a backward elimination process (statistical analysis and results are reported in Figure A2). The residuals of the model were normally distributed (Anderson-Darling *p* = 0.695) and the adjusted-R^2^ was 0.9662 with a not significant lack of fit (*p* = 0.212). The response obtained did not depend on cysteine concentration in the design space and had a maximum with a short warming time and a sulfuric acid/water ratio close to 2.1 (Figure 2A).

The input parameters were optimized in order to maximize the fucose ΔABS while minimizing the corresponding glucose ΔABS in order to maintain the selectivity of the method for methyl-pentoses with respect to hexoses eventually present in the same polysaccharide sample. The conditions that maximize the desirability function were a sulfuric acid/water ratio of 2.1, a warming time of 5 min, and a cysteine concentration of 32 mM (Figure 2).

In a separate experiment the color development kinetic was checked for a 2.1 ratio H_2_SO_4_/water with 5 min of warming time for multiple cysteine concentrations and it became quite stable between 5–10 min independently from the cysteine concentration, starting then to diminish significantly.

Keeping constant the H_2_SO_4_/water ratio of the reaction, the direct addition of sulfuric acid (instead of its water mixture) enhances the volume of the sample that can be used, resulting in an enhancement of the method sensitivity.

Also, it has been verified that the following substances do not produce signal in the Dische assay: hexose (glucose tested up to 100 μg/mL, galactose and mannose tested up to 40 μg/mL), 2-aminohexose (N-acetylglucosamine tested up to 10 μg/mL), uronic acid (glucuronic acid tested up to 40 μg/mL), 3,6-dideoxy hexose (tyvelose tested up to 20 μg/mL), KDO (3-deoxy-D-manno-oct-2-ulosonic acid tested up to 20 μg/mL), protein (BSA tested up to 100 μg/mL) and DNA (tested up to 25 μg/mL) while it has been found interference for *sodium azide* even at 0.05% concentration in solution.

### 2.2. Dische Applied to S. flexneri 6 Serotype OAg

For the quantification of *S. flexneri* serotype 6 OAg, the monosaccharide Rha was chosen for building the calibration curve.

#### 2.2.1. Standard Linearity

After a first screening in the range 0.05–10 μg/mLof Rha, the linearity of the method was successfully assessed in the range of 0.2–2 μg/mL.

A regression analysis on the data generated showed a significant linear model, the lack of fit was not significant, and the residuals were normally distributed (Anderson Darling *p* = 0.081) (Figure A3). The calibration curve range 0.2–2 μg/mL of Rha corresponds to 0.41–4.1 μg/mL of *S. flexneri* 6 OAg considering the repeating unit molecular weight.

In the optimized conditions identified, the achieved linearity in the range of 0.2–2 μg/mL of Rha allowed a 10-fold increment of the sensitivity respect to the original method.

#### 2.2.2. Reproducibility

The intermediate precision (defined as the variability among different sessions), the repeatability and the contribution to the variability of the analysis session, expressed as coefficient of variation (CV), are reported in Table 1 for three different concentration levels along the calibration curve (statistical analysis reported in Figure A4): 0.41–2.05–4.10 µg/mL of *S. flexneri* 6 OAg (corresponding to 0.2–1–2 µg/mL as Rha content).

#### 2.2.3. Sample Linearity

The regression model for the sample values measured against the theoretical concentrations (statistical analysis reported in Figure A5) was significant, with lack of fit not significant, and the residuals normally distributed. The intercept was not significantly different from zero and the slope 95% confidence interval (CI) was 1.0775–1.1560.

This means that by using the Rha as a calibration curve to quantify *S. flexneri* 6 OAg content, there is an average over-estimation of 11%.

### 2.3. OAg Hydrolysis Followed by HPAEC-PAD

#### 2.3.1. Polysaccharide Hydrolysis Conditions

For quantification of the monomers constituting a polysaccharide by HPAEC-PAD, hydrolysis conditions that allow maximum release of the monomers without their degradation need to be identified. Recently, it was verified that acid hydrolysis with concomitant use of TFA and HCl allowed the release and quantification of 2-amino uronic acids [20]. We tried to extend the same approach to the quantification of GalA contained in *S. flexneri* 6 OAg.

Starting from acid hydrolysis conditions optimized for 2-amino uronic acid (HCl 8 M + TFA 10% at 80 °C), we tested in a kinetic of hydrolysis the commercial GalA as a reference and the *S. flexneri* 6 OAg. Chromatographic conditions used were appropriate for elution of negatively charged sugars. The results of this analysis showed that the GalA standard was destroyed by 50% after only one hour of heating time. For the OAg sample, we observed formation of GalA and of an unknown peak both decreasing over time.

The hydrolysis was repeated in milder conditions, decreasing the temperature down to 60 °C and changing HCl (1, 4 and 8 M) and TFA (10 and 20%) concentrations and hydrolysis time (1 and 2 h). We found that the GalA was unstable in all conditions tested.

After that a DoE experiment was performed using milder hydrolysis conditions to better investigate the different parameters involved and their interaction. HCl concentration, TFA concentration, time and temperature of hydrolysis were the factors evaluated in the range 4–8 M, 0–20% *v*/*v*, 1–4 h, and 40–60 °C, respectively (Table A2).

From the results, no statistically significant model was identified for GalA formation.

Among the tests performed, a recovery of 96% of GalA standard solution was obtained after hydrolysis at 60 °C, with 0% TFA, HCl 4 M, for 60 min. The same conditions did not allow for the release of the GalA from the *S. flexneri* 6 OAg. However, looking at the chromatograms derived from *S. flexneri* 6 OAg hydrolyses, we identified the consistent presence of un unknown peak at 6.5 min with a reasonable stability in the hydrolysis conditions tested.

Considering the structure of *S. flexneri* 6 OAg, we hypothesized the formation of the dimer GalA-GalN and decided to verify its formation also by performing the hydrolysis with only TFA, using conditions commonly reported for Rha release [22]. A kinetic of hydrolysis was performed with TFA 2 M at 100 °C, and reaction mixtures were analyzed with chromatographic conditions to detect both Rha and negatively charges species. Both analyses confirmed the maximum release of Rha and of the unknown species after 2 h (Figure A6). It is worth noting that, starting from 2 h of hydrolysis, the Rha peak showed a small shoulder at a higher retention time, verified to be galactosamine (GalN). The peak increased over time and became very clear after 6 h (Figure A7). Tests were performed to improve the separation of the two peaks [23] without success. In parallel, while the area of Rha remained stable up to 6 h, the area of the unknown peak slightly decreased.

#### 2.3.2. Characterization of the Unknown Peak Coming from Acid Hydrolysis (Dimer GalA-GalN)

**Figure 3:**
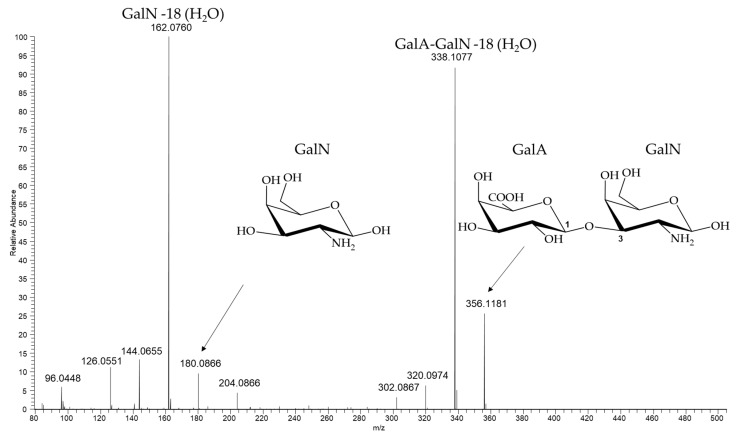
ESI MS spectrum, positive ion mode ([M + H] + ions), of *S. flexneri* 6 OAg hydrolyzed with TFA 2 M at 100 °C for 2 h, indicating formation of the dimer GalA-GalN.

The reaction mixture coming from the identified hydrolysis conditions (2 h TFA 2 M 100 °C) was analyzed by ^1^H NMR in comparison to Rha, GalA and GlaN monomers (Figure A8). Signals corresponding to free Rha (α and β configuration) were identified, but no presence of free GalA and GalN was detected. However, anomeric signals of GalA (β configuration only) and GalN (α and β configuration) resulted to be slightly shifted respect to corresponding standard monomers. This finding supported possible formation of the dimer GalA-GalN, which presence was finally confirmed by MS analysis (Table 2, Figure 3). The MS also revealed presence of GalN, also detected in HPAEC-PAD in small amounts.

The linkage 1→3 between β-GalA and α-β-GalN in the dimeric structure formed by hydrolysis of the PS was verified using a combination of HMBC, HSQC spectra (Figure A9) and a COSY spectrum (Figure A10).

**Figure 4:**
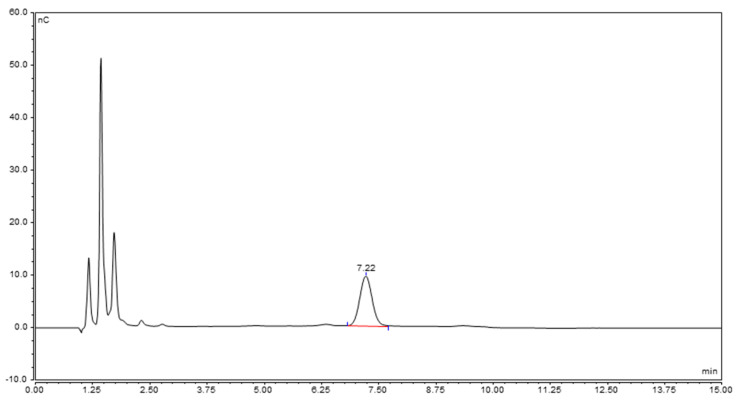
HPAEC-PAD chromatogram of the dimer GalA-GalN obtained after hydrolysis of *S. flexneri* 6 OAg, eluted in CarboPac PA1 column with NaOH 400 mM as eluent.

**Figure A1:**
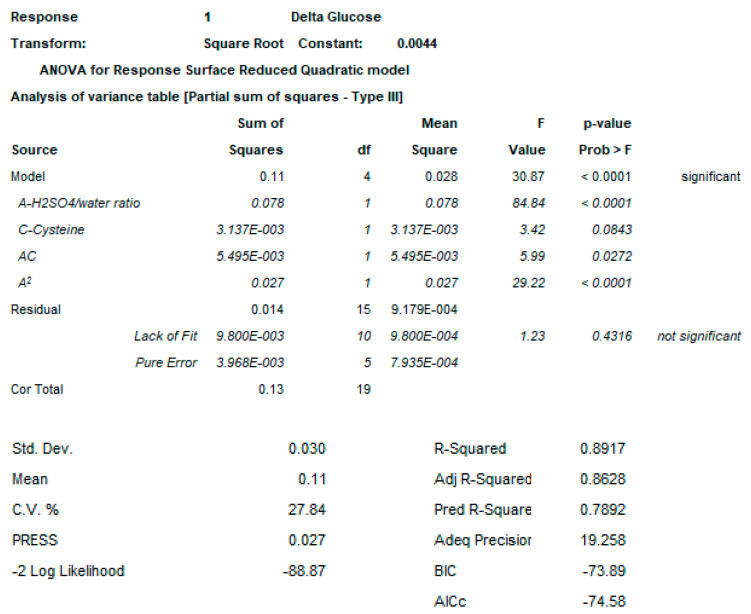
DoE statistical analysis for glucose response surface in Dische colorimetric method.

**Figure A2:**
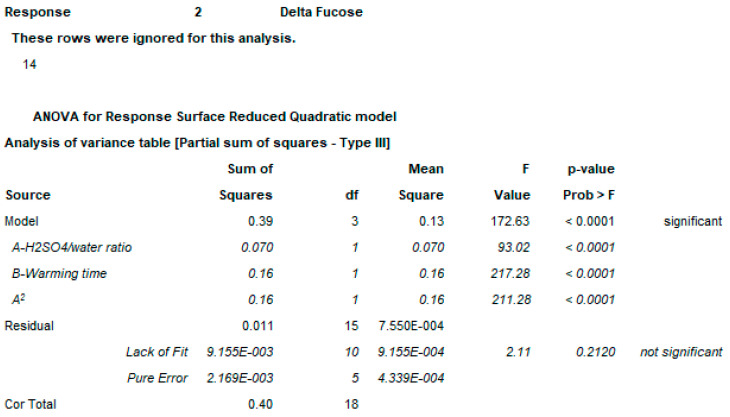

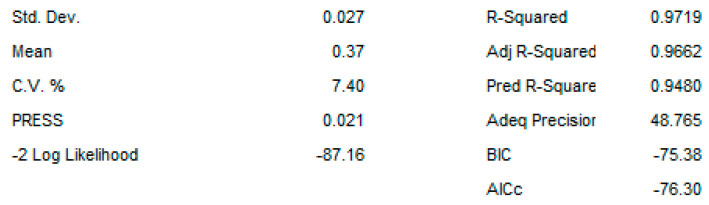
DoE statistical analysis for fucose response surface in Dische colorimetric method.

**Figure A3:**
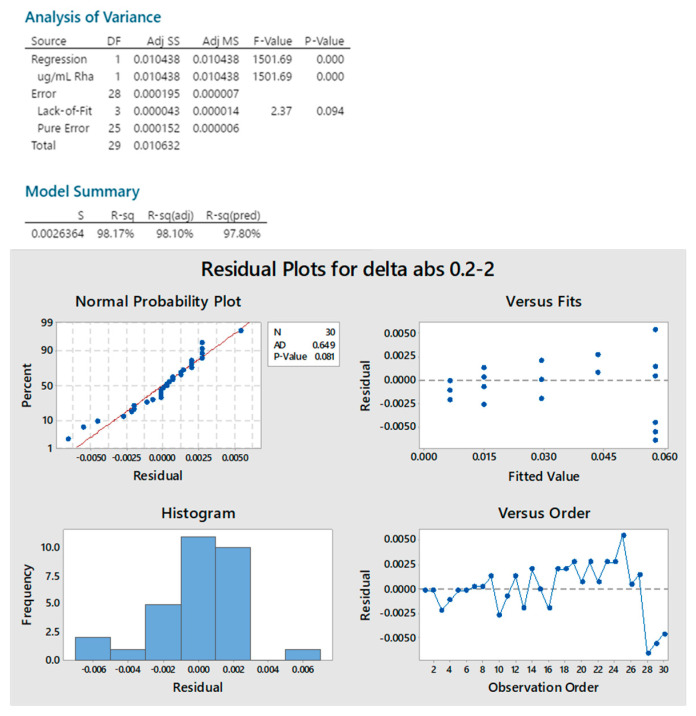
Standard Linearity determination for Dische colorimetric method: ANOVA on 5 replicates of the calibration curve.

Based on the results obtained, we decided to perform hydrolysis with *S. flexneri* 6 OAg as standard, with TFA 2 M at 100 °C for 2 h, detecting the dimer by HPAEC-PAD (Figure 4).

#### 2.3.3. Hydrolysis Yield Determination by qNMR

Using quantitative NMR (qNMR), we measured that the hydrolysis conditions identified lead to the dimer GalA-GalN with a yield of 85%.

#### 2.3.4. Standard Linearity

After a first screening in the range 0.1–10 μg/mL for standard *S. flexneri* 6 OAg, the linearity of the method was successfully assessed in the range of 0.56–5 μg/mL.

A regression analysis on the data generated showed a significant linear model and a not significant lack of fit (*p* = 0.694) with the residuals normally distributed (Anderson-Darling *p* = 0.092) (Figure A11).

#### 2.3.5. Reproducibility

The intermediate precision (defined as the variability among different sessions), the repeatability and the contribution to the variability of the analysis session, expressed as coefficient of variation (CV), are reported in Table 3 for three different concentration levels along the calibration curve (statistical analysis reported in Figure A12).

#### 2.3.6. Sample Linearity

The regression model for the sample values measured against the theoretical concentrations (plot reported in Figure A13, statistical analysis reported in Figure A14) was significant with a slope value close to one and an intercept value close to zero. However, there was a significant lack of fit (*p* = 0.038). In our experience, when very low CV values are found for repeatability (Table 3), it is easy to demonstrate a significant lack of fit in the ANOVA that has no practical consequences on the assay. As a confirmation, the significance of an eventual second order coefficient for the regression was checked (Figure A15), but resulted to be not significant (*p* = 0.070).

Moreover, the linear regression analysis (Figure A13) evidenced a non-normal residual distribution (Anderson-Darling *p* = 0.024) with heteroscedasticity that agrees with the quite constant CV found for reproducibility at the different concentrations along the calibration curve. Looking in depth to the analysis of residuals (Figure A16), the shape of their distribution was highly symmetric (mean and median CI quite completely overlapping), the result of the Anderson-Darling normality was due mainly to kurtosis. Using the Kolmogorov-Smirnov test as alternative to the Anderson-Darling one, the residuals did not result non normally distributed (*p-*value > 0.150).

Finally, by checking the sample linearity in each of the six analysis sessions (Figure A17 and Figure A18), only one out of six showed a significant lack of fit and all of them had the residual normally distributed, the linear coefficient not significantly different from 1 and the intercept not significantly different from zero (Table A3).

Based on all of these findings and considering the purpose of the sample linearity analysis, the results obtained were satisfactory.

#### 2.3.7. Accuracy (Spike Recovery)

The accuracy of the method was evaluated by spike recovery on a sample of *S. flexneri* 6 OAg co-conjugated to CRM_197_ [17] and the results are reported in Table 4 for three different OAg spike concentration levels (single spike recovery values and analysis are reported in Table A4 and Figure A19).

## 3. Discussion

Many vaccines in development against *Shigella* are OAg based [9]. In particular, the research is active to develop multicomponent vaccines with broad protection against different *Shigella* serotypes, and *S. flexneri* 6 has been proposed as one of the components of these formulations [12].

Here, we have optimized two different methods for *S. flexneri* 6 OAg quantification that are critical for vaccine release and to assess its vaccine stability over time. Two colorimetric methods had been previously used at this scope [14,15,16]. We have further improved one of them, the Dische colorimetric assay, making it more user friendly (reactions are performed directly in the cuvette with straight addition of sulfuric acid) and more sensitive (from 2 to 0.2 μg/mL of Rha used to build the calibration curve). The sensitivity was increased of around 10-fold, always maximizing the response to methyl-pentoses, while minimizing the response to hexoses.

Also, we have developed a novel method specific for *S. flexneri* 6 OAg based on acid hydrolysis followed by HPAEC-PAD analysis. The method had issues related to instability of the GalA monomer released in certain conditions (by using it as analyte), or of analyte separation by chromatography (by using neutral sugars Rha or GalN as analytes). We have overcome these issues by identifying hydrolysis conditions to form the stable dimer GalA-GalN that has been fully characterized by MS and mono- and bi-dimensional NMR analyses and that can be detected using chromatographic conditions free of possible interferences.

Both methods have been characterized for their performance, that based on our experience was in line with the performance of other polysaccharide quantification methods used for vaccine release.

The two methods are similar in their sensitivity, with both able to detect concentrations less than 1 µg/mL. The Dische colorimetric method is quite fast and particularly useful as a method for in process control. The HPAEC-PAD method requires longer time for analysis but has the advantage to be specific for *S. flexneri* 6.

For the identification of the optimal assay condition, we made use of statistical tools, e.g., DoE, that allow for the recognition of the critical method parameters and their optimal combination to maximize the response desired. This is much more efficient and fast with respect to investigation of the effect of one parameter at time.

These improved methods will facilitate characterization of vaccines containing *S. flexneri* 6 OAg as active ingredient. Furthermore, the Dische colorimetric method is readily applicable to other polysaccharides containing methyl-pentoses, like fucose and Rha, such as *Shigella*, *Salmonella* OAg and *Klebsiella* capsular polysaccharides. The HPAEC-PAD method though more specific could be extended to other polysaccharides containing uronic acids, such as *Klebsiella* capsular polysaccharides.

## 4. Materials and Methods

### 4.1. Materials

*S. flexneri* 6 OAg was purified and characterized as previously described [17]. Different lots at average molecular weight of 22 kDa (as estimated by HPLC-SEC analysis) were used, identity was confirmed by ^1^H NMR and the purity in terms of residual proteins and DNA resulted similar for all lots. *S. flexneri* 6 OAg glycoconjugate was produced with OAg of same molecular weight by random chemistry as reported in [17].

Cysteine, Glucose, Galactose, Mannose, Fucose, Galacturonic acid, Rhamnose, Galactosamine, N-acetylglucosamine, KDO, BSA, sodium azide, trifluoroacetic acid, hydrochloric acid were purchased from Sigma-Aldrich (Burlington, MA, USA). Tyvelose was purchased from Toronto Research Chemicals (Toronto, ON, Canada). Sulfuric acid 95–98% was purchased from Merck (Whitehouse Station, NJ, USA). Sodium Hydroxyde 50% was burchased from JT Baker (Radnor, PA, USA).

### 4.2. Dische Colorimetric Method

#### 4.2.1. DoE

Each sample was prepared in a tube with 200 μL of glucose or fucose (50 μg/mL in water) adding in sequence water and ice-cold H_2_SO_4_ to a total volume of 1.5 mL (as reported in Table A1). The mixture was mixed by vortexing and placed in a preheated thermoblock at 100 °C for the required time, then cooled in ice for further 10 min. 0.5 mL of the mixture was transferred in a cuvette and ABS at 396 and 427 nm were read (to be used as blank). The required amount of cysteine 1 M was added to the remaining 1 mL of the sample, mixed by vortexing and kept 10 min at room temperature for color development. The sample was then transferred in a cuvette to read 396 and 427 nm ABS. For each sample the ΔABS was calculated using the following equation (DoE response).

$$\mathrm{ABS}=({\mathrm{ABS}}_{396}^{post\;cys}-{\mathrm{ABS}}_{396}^{pre\;cys})-({\mathrm{ABS}}_{427}^{post\;cys}-{\mathrm{ABS}}_{427}^{pre\;cys})$$

#### 4.2.2. Optimized Quantification Method

To each standard/sample (500 µL in an Eppendorf tube), 1050 µL of ice cooled sulfuric acid are added and the tubes are mixed by vortexing. The tubes are then heated at 100 °C in the thermoblock for 5 min and finally cooled in ice for 10 min. One milliliter from each standard/sample cooled tube is transferred into disposable cuvettes (code 1938, Kartell, Milan, Italy) and the ABS 427 and 396 nm are read to be used as blank. Thirty-two microliters of cysteine 1 M are then added to each cuvette, the cuvettes are sealed with cap (code 759240, Brandtech, Essex, CT, USA) and mixed by vortexing. After 10 min at room temperature, the ABS 427 and 396 nm are read. For each standard and sample replicate, the ΔABS value is calculated with the formula reported above, subtracting from each sample/standard its own blank. A linear regression is performed on ΔABS Vs standard concentrations and the sample methyl-pentoses content is then calculated.

The calibration curve is performed in single replicate, while the samples are assayed in duplicate, averaging the results. Rhamnose standard solution is used to build the calibration curve.

#### 4.2.3. Standard Linearity

To assess the linearity of the method, five different replicates of the Rha calibration curve were tested at concentrations of 0.2–0.5–1–1.5–2 μg/mL. The preparation order of the standards and their ABS reading order were randomized.

A linear regression analysis on the data was performed.

#### 4.2.4. Reproducibility and Sample Linearity

To assess the reproducibility and sample linearity for the analysis of *S. flexneri* 6 OAg sample [17], a total of six analysis sessions was performed on six different days.

In each analysis session, the sample solution was diluted in order to obtain three replicates for the 0.41, one for the 1.03, three for the 2.05, one for the 3.08 and three for the 4.10 μg/mL concentration levels (scheme reported in Table A5). In each session, sample preparation order and analysis run order were randomized.

**Figure A4:**
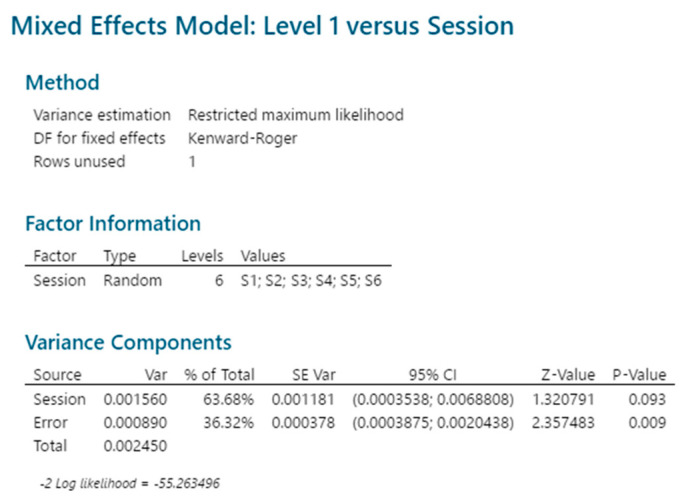

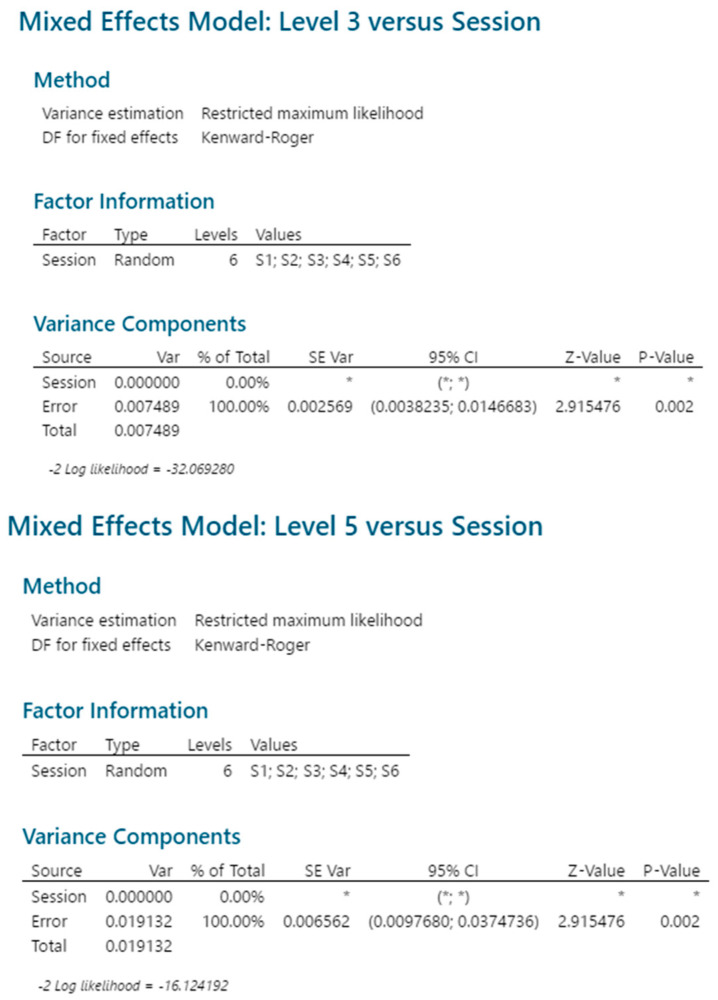
Variance component analysis at three different *S. flexneri* 6 OAg concentration levels for reproducibility in Dische colorimetric method; * not applicable.

**Figure A5:**
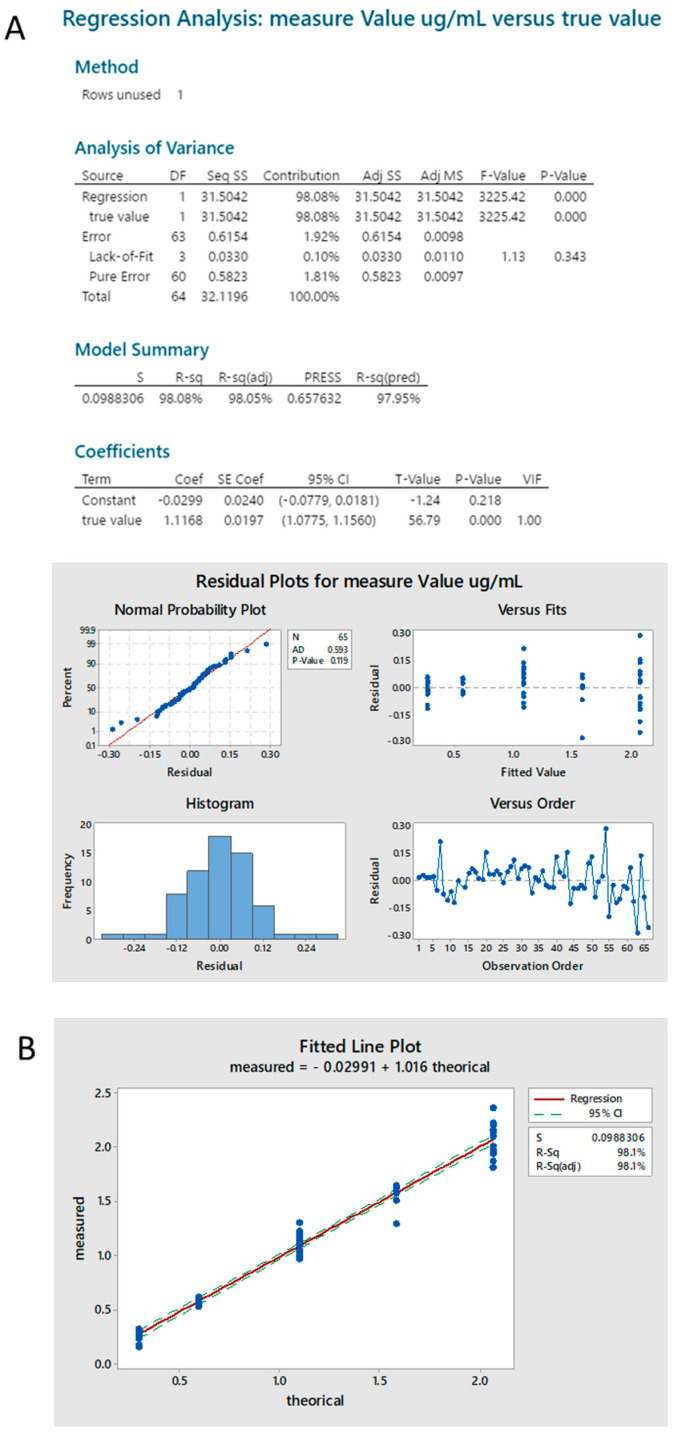
Sample Linearity determination for quantification of *S. flexneri* 6 OAg with Dische colorimetric method: (**A**) ANOVA on 5 replicates for the calibration curve; (**B**) calibration curve plot.

**Figure A6:**
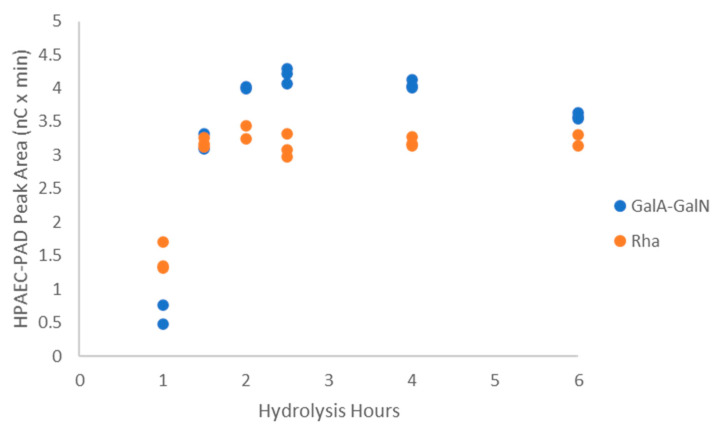
Kinetic of hydrolysis of *S. flexneri* 6 OAg with TFA 2 M at 100 °C: HPAEC-PAD peak area for Rha and GalA-GalN dimer Vs hydrolysis time is reported.

**Figure A7:**
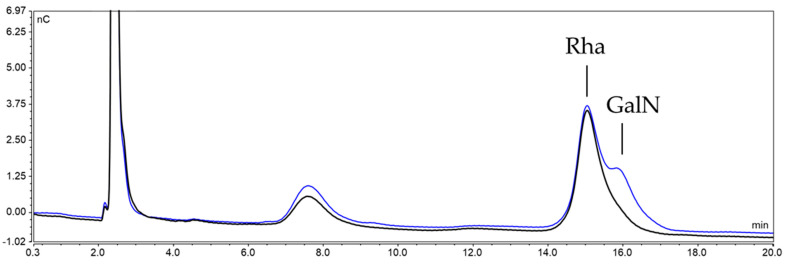
Overlay HPAEC-PAD chromatograms of *S. flexneri* 6 OAg hydrolyzed with TFA 2 M at 100 °C for 2 h (black line) or 6 h (blue line).

**Figure A8:**
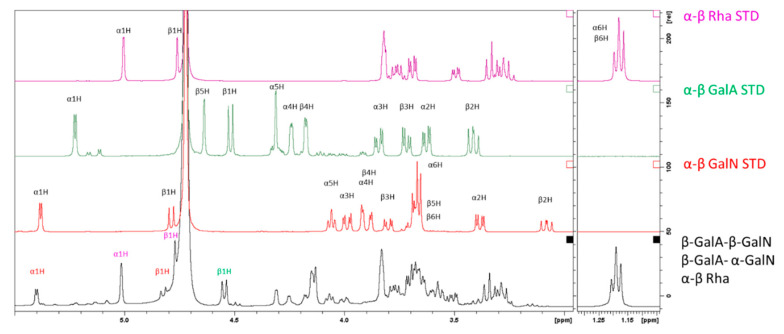
^1^H NMR spectrum of the hydrolyzed *S. flexneri* 6 OAg containing dimer GalA-GalN (black) in comparison with the standard sugars of Rha (violet), GalA (green) and GalN (red).

**Figure A9:**
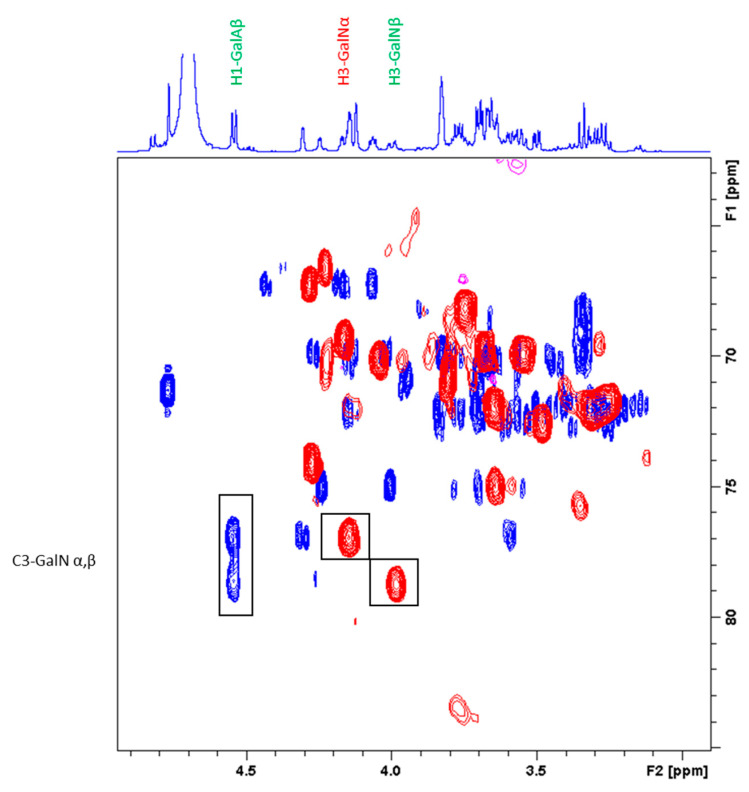
Overlap of ^1^H-^13^C HMBC NMR spectrum (Blue) and ^1^H-^13^C HSQC NMR spectrum (Red-Pink) of *S. flexneri* 6 OAg after TFA hydrolysis.

**Figure A10:**
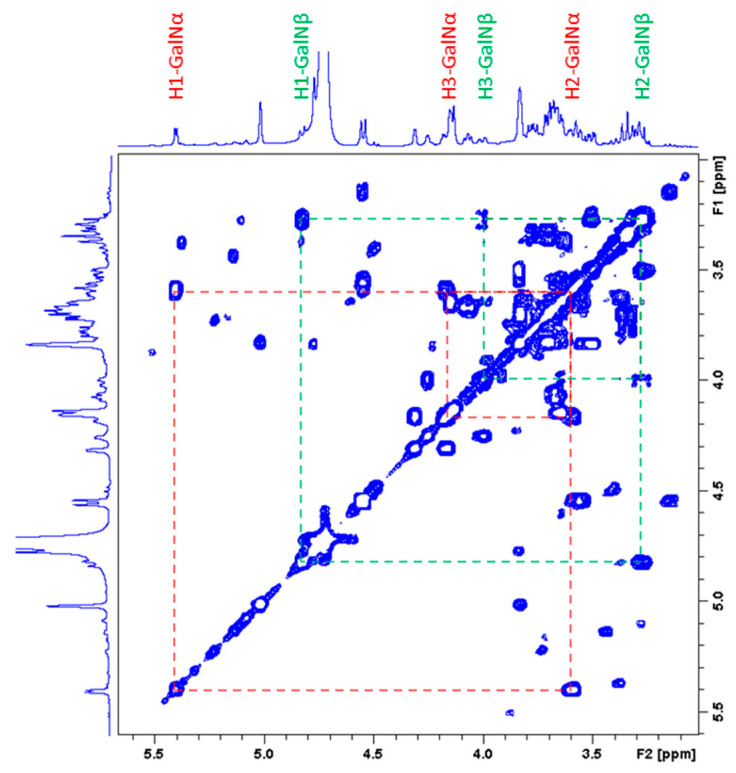
^1^H-^1^H COSY NMR spectrum of *S. flexneri* 6 OAg after TFA hydrolysis.

**Figure A11:**
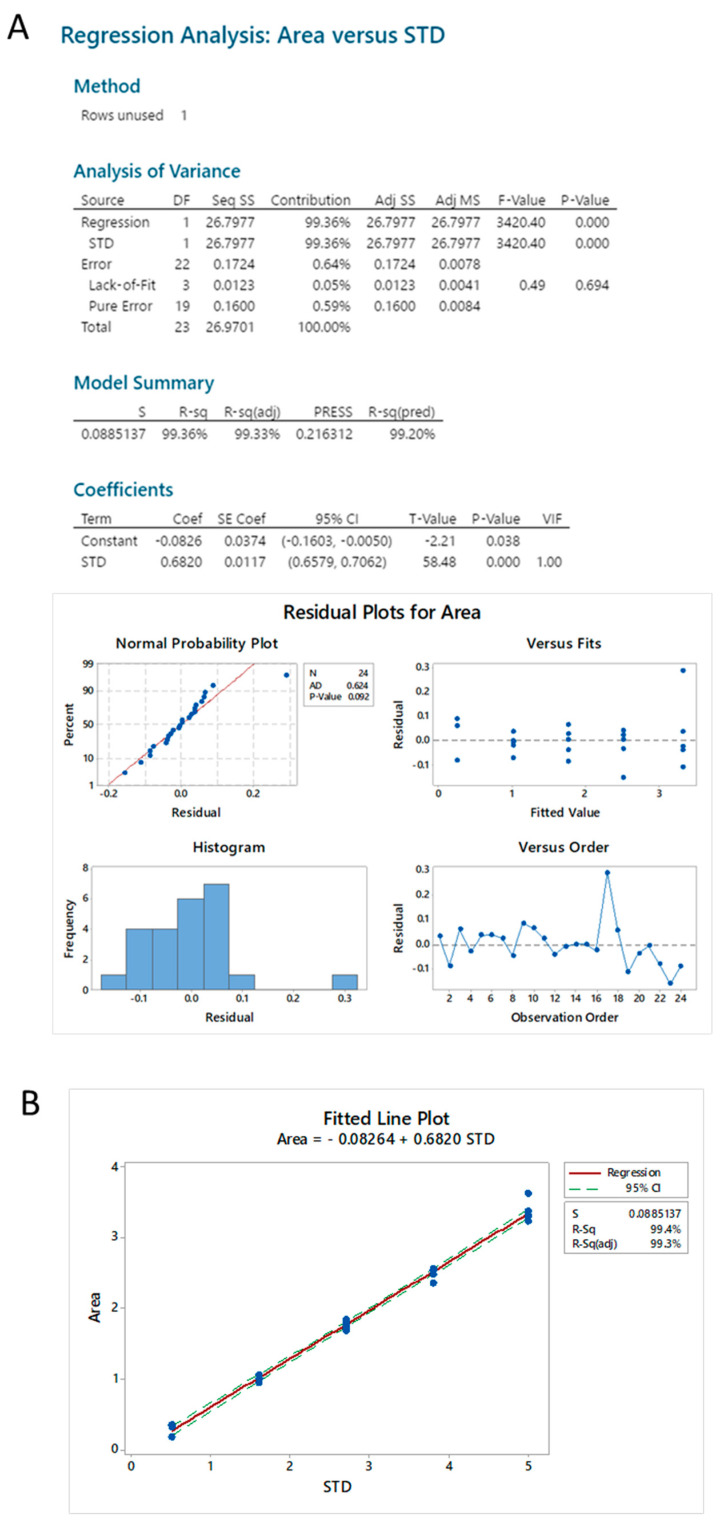
Standard Linearity determination for quantification of *S. flexneri* 6 OAg by acid hydrolysis followed by HPAEC-PAD: (**A**) ANOVA on 5 replicates for the OAg calibration curve; (**B**) calibration curve plot.

Reproducibility has been calculated using a variance component analysis (REML, Figure A4) on data generated in six analysis sessions respectively at 0.41, 2.05 and 4.10 μg/mL concentration levels.

Sample linearity was determined on all data generated in the six analysis sessions, with an analysis of regression plotting the values measured against the theoretical concentrations calculated by sample dilution. The true concentration of the more concentrated sample was assigned from the average of all its measurements for assessing sample linearity and reproducibility (18 values).

### 4.3. OAg Hydrolysis Followed by HPAEC-PAD

#### 4.3.1. Optimized Analysis Conditions (GalA-GalN Dimer Quantification)

Using an Xstream Electronic Pipettor (Eppendorf, Hamburg, Germany), 150 μL of TFA 8 M are added to 450 μL of solution containing sample/standard in a 2 mL screw cap vial; the lid is closed, and the content is mixed by vortexing. The hydrolysis vials containing samples/standards are maintained in a preheated oven at 100 °C for 2 h.

After hydrolysis, the vials were removed from the oven and cooled to room temperature. The content of the vials was then evaporated to dryness using a centrifugal evaporator.

After drying, the content of each vial is re-dissolved in 450 μL of water and accurately mixed by vortexing. The content of each vial is transferred into a 0.2 filtration 96-well plate, placed over a 96 conical BTM plate and centrifuged (Allegra X-15 with SX4750 swinging-bucket rotor and 393070 microplate carrier; Beckmann-Coulter, Brea, CA, USA) at 524 rcf for 3 min to collect filtered samples.

The plate containing filtered sample/standard solutions is covered with the pre-slit 96-well cap and put in the HPAEC-PAD autosampler compartment.

Each sample is assayed in triplicate, averaging the results, and two different calibration curves were run prior to and after the samples were complete. Standardized *S. flexneri* 6 OAg was used to build the calibration curves.

#### 4.3.2. Chromatographic Conditions:

The chromatographic runs were performed on ICS5000 or ICS-3000 equipped with Chromeleon 7.2 (Thermo, Waltham, MA, USA) using the pulsed amperometric mode with gold working electrode and Ag/AgCl reference electrode applying standard quad carbohydrate waveform.

- Neutral sugar determination: 5 μL injection volume, column and detector temperature 25 °C, CarboPac PA10 4 × 50 mm guard column connected in series with PA10 4 × 250 mm column (Thermo). Eluent program: NaOH 18 mM × 30 min (flow rate 0.6 mL/min), NaOH 500 mM × 10 min (flow rate 1.6 mL/min), NaOH 18 mM × 38 min (flow rate 1.6 mL/min).
- Uronic acid determination (analysis conditions reported in Giannelli et al. [20] for 2-aminouronic acids): 25 μL injection volume, column and detector temperature 25 °C, CarboPac PA1 4 × 50 mm guard column connected in series with PA1 4 × 250 mm column (Thermo). Eluent program: NaOH 400 mM × 15 min (flow rate 1.5 mL/min).

#### 4.3.3. DoE

Each hydrolysis test was performed in parallel on vials with 2.4 µg *S. flexneri* 6 OAg (dried) and on vials with 0.6 µg GalA (dried), in both cases resuspended in 1 mL of the acid hydrolysis mixture. For the DoE runs acid composition, hydrolysis time and temperature used are detailed in Table A2.

After the hydrolysis, samples were dried and stored at 4 °C until the analyses were performed. For the analysis, samples were resuspended in 300 µL of water and analyzed by HPAEC-PAD in a single analysis session where the injection order followed the same randomization scheme used for the hydrolysis (Table A2).

A D-optimal split plot design was used with temperature as hard to change factor (range 40–60 °C) and TFA (0–20%), HCl (4–8 M) and hydrolysis time (60–240 min) as easy to change factors.

Eleven temperature groups in total, including six center point groups with a group size of three were performed, resulting in a total of 44 runs.

#### 4.3.4. Mass Spectrometry (MS)

High resolution mass spectra were recorded on Q-Exactive plus (Thermo) by direct infusion of the sample at 10 μL/min. One milligram of polysaccharide was hydrolyzed in the optimized conditions (TFA 2 M, 2 h, 100 °C), dried, and resuspended at 200 μg/mL final concentration in 80% acetonitrile/20% water. The following parameters were used: scan range 80–2000 *m*/*z*; resolution 70,000; positive ion mode; sheath gas flow rate 5; auxiliary gas flow rate 1; sweep gas flow rate 0; spray voltage 3.8 kV; capillary temperature 100 °C; S-lens RF level 60; and aux gas heater 40 °C.

#### 4.3.5. Nuclear Magnetic Resonance (NMR) Spectroscopy

Samples (~1 mg polysaccharide) were lyophilized and exchanged twice with 99.9% deuterium oxide (Sigma-Aldrich), then dissolved in 500 μL of D_2_O and introduced into a 5 mm NMR tube (Sigma-Aldrich) for data acquisition. 1D ^1^H and 2D COSY NMR spectra were recorded at 298 K with an Avance III 400 spectrometer (Bruker, Billerica, MA, USA) using standard pulse sequences. ^1^H NMR spectra were recorded at 400 MHz; chemical shift values are reported in ppm; and the solvent peak for D_2_O was calibrated at 4.79 ppm.

The *S. flexneri* 6 OAg hydrolyzed (1 mg) was also analyzed using an AEON AVANCE III 600 MHz spectrometer (Bruker) equipped with a high-precision temperature controller using a 5 mm QCI CryoProbe. The probe temperature was set at 298 K. The solvent peak for D_2_O was calibrated at 4.70 ppm. 1D ^1^H and 2D HSQC, HMBC NMR were recorder. The HSQC experiment was optimized for J = 145 Hz (for directly attached ^1^H-^13^C correlations), the HMBC experiment was optimized for a coupling constant of 6 Hz (for long-range ^1^H-^13^C correlations).

For quantitative NMR (qNMR) spectra, a solution of *S. flexneri* 6 OAg was transferred into two screw cap vials and dried to have respectively 2.4 mg and 0.4 mg of polysaccharide for hydrolysis and reference sample in each vial. The sample subjected to hydrolysis was resuspended in water and treated with a final mixture of TFA 2 M for 2 h at 100 °C, then dried. The dried polysaccharide was used as reference and the dried hydrolyzed polysaccharides were resuspended in D_2_O (500 μL). The polysaccharide in the reference vial was de-O-acetylated adding 35 μL of NaOH 4 M in D_2_O and warming at 37 °C for 2 h. 150 μL of maleic acid standard solution (350 μg/mL) was added to both samples.

Spectra were acquired using a total recycle time to ensure a full recovery of each signal (5 × longitudinal relaxation time T1).

The hydrolyzed sample was quantified using the ratio between the dimer GalA H-1β signal and the maleic acid internal standard. The de-O-acetylated polysaccharide was quantified using the ratio between the methyl signal of both rhamnoses and the maleic acid internal standard. The hydrolysis yield was estimated by calculation as a ratio between the two quantifications.

#### 4.3.6. Standard Linearity

To assess the linearity of the method, five different replicates of the *S. flexneri* 6 OAg calibration curve were tested at concentrations of 0.56–1.67–2.78–3.89–5 µg/mL. The preparation order of the standards and their chromatographic run order were randomized.

A linear regression analysis on the data was performed.

#### 4.3.7. Reproducibility and Sample Linearity

To assess the reproducibility and sample linearity for the analysis of *S. flexneri* 6 OAg sample, a total of six analysis sessions was performed in six different days.

In each analysis session, the sample solution was diluted in order to obtain three replicates for the 1.00, one for the 2.00, three for the 2.89, one for the 3.50 and three for the 4.50 μg/mL concentration levels (scheme reported in Table A5). In each session, sample preparation order and analysis run order were randomized.

**Figure A12:**
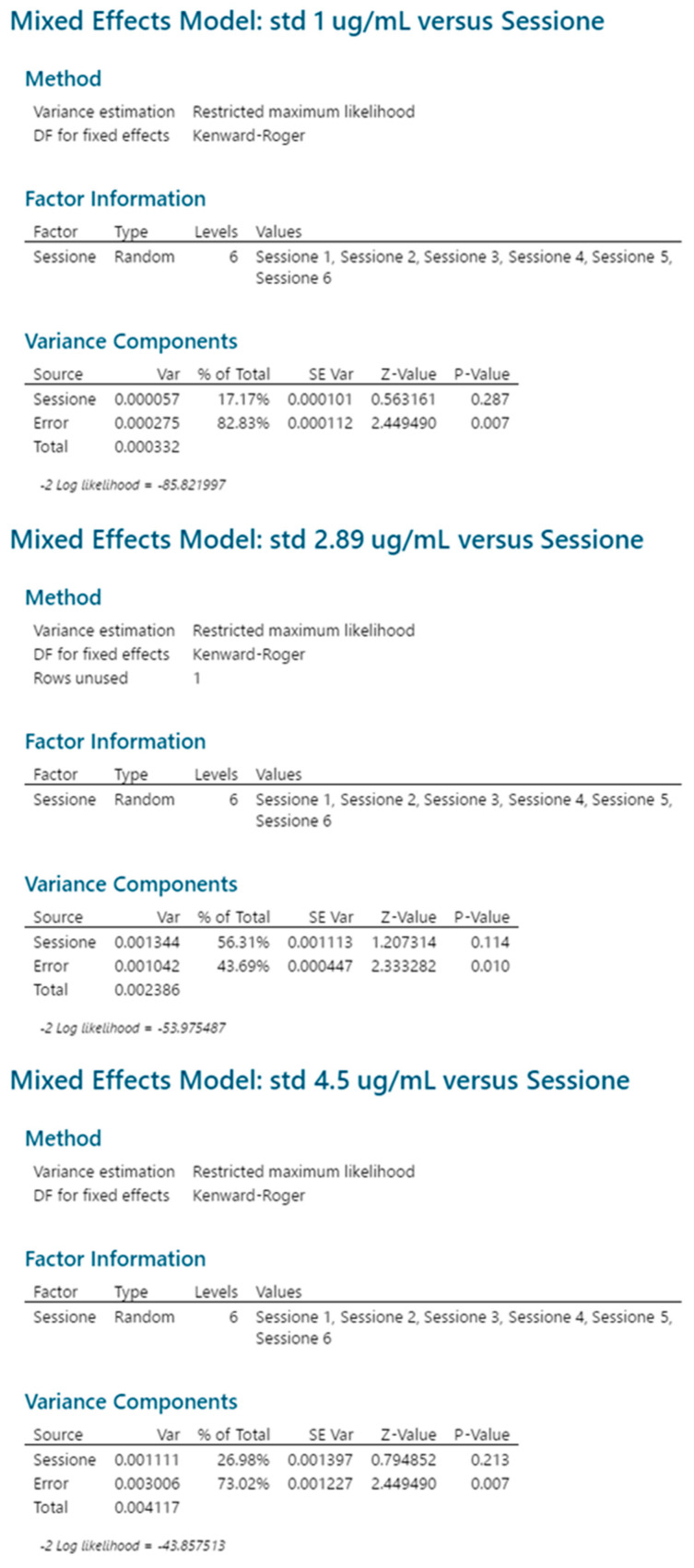
Variance component analysis at three different concentration levels for reproducibility assessment of HPAEC-PAD analysis.

**Figure A13:**
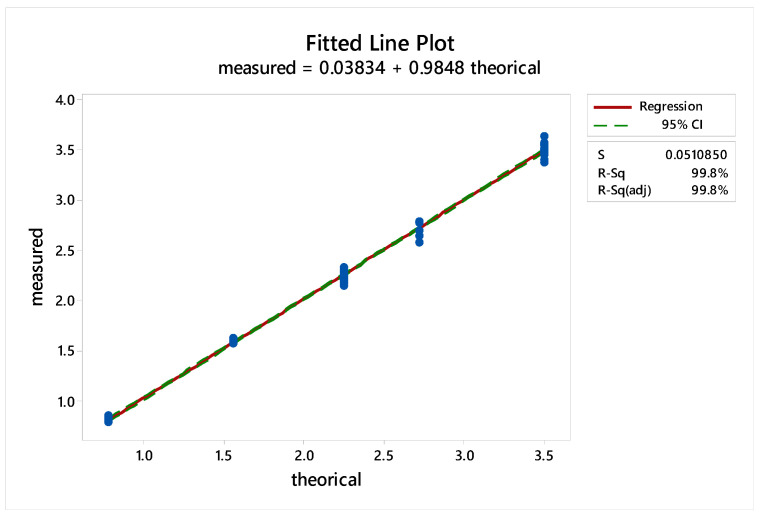
Sample Linearity plot for quantification of *S. flexneri* 6 OAg by HPAEC-PAD.

**Figure A14:**
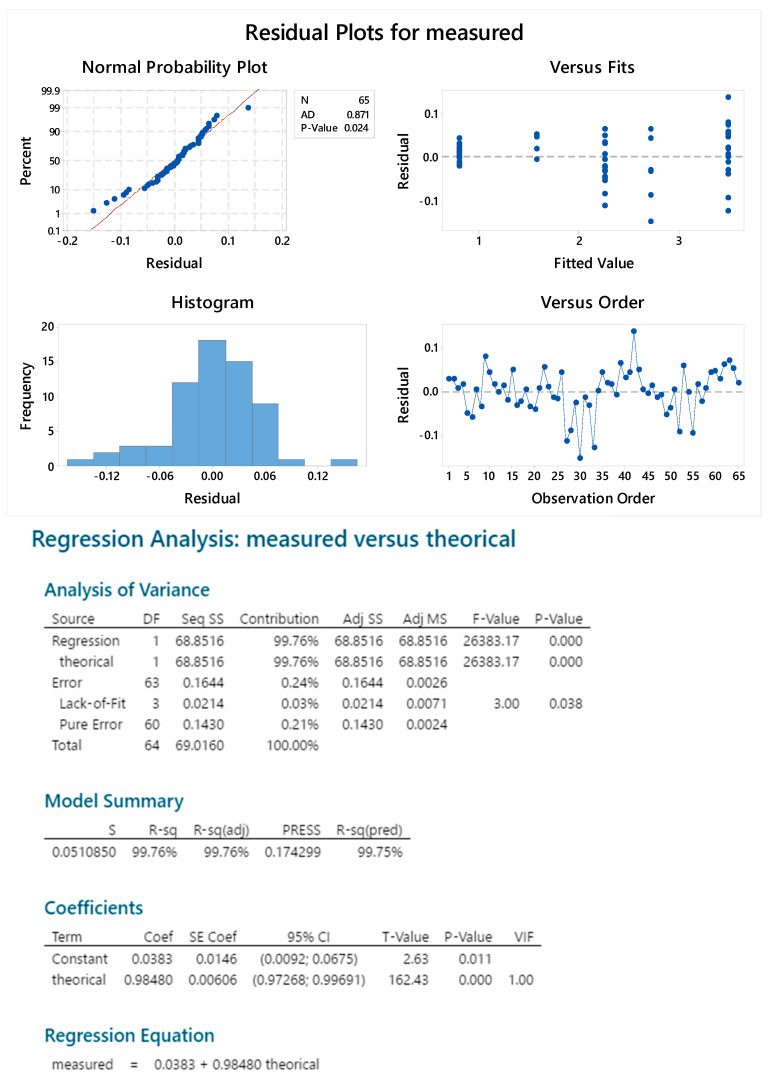
Linear regression analysis for sample Linearity determination for quantification of *S. flexneri* 6 OAg by HPAEC-PAD (data from six analysis sessions together).

**Figure A15:**
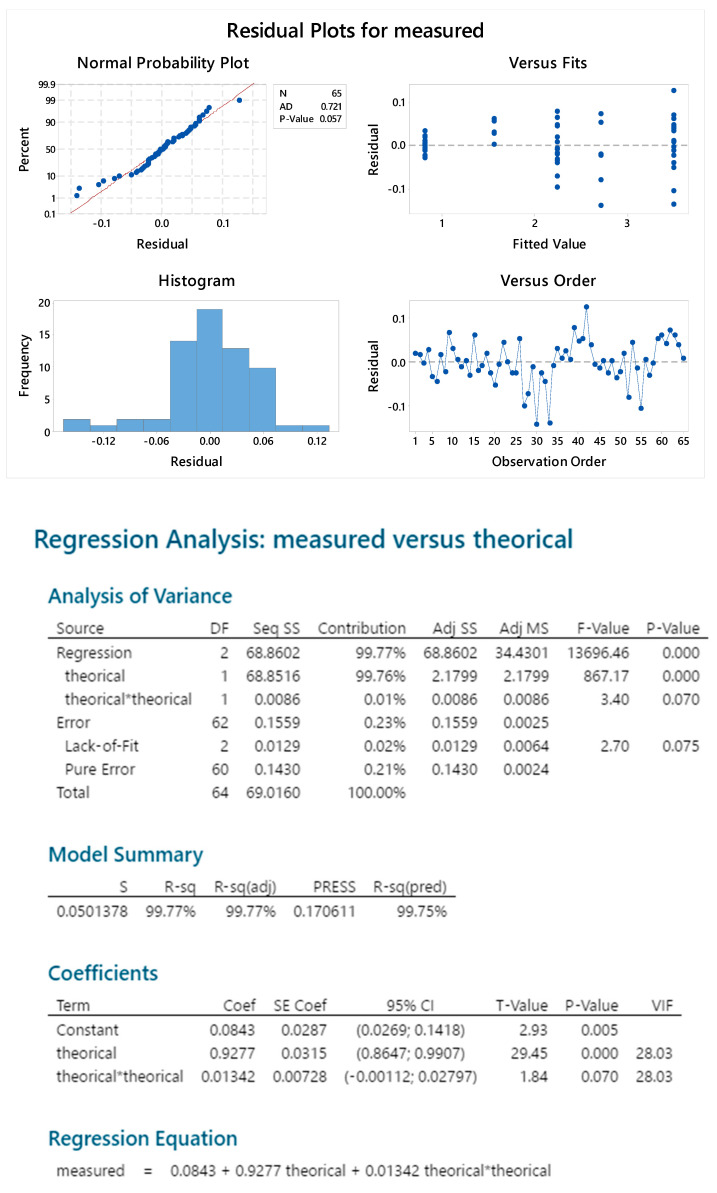
Quadratic regression analysis for sample linearity in of *S. flexneri* 6 OAg assay by HPAEC-PAD (data from six analysis sessions together).

**Figure A16:**
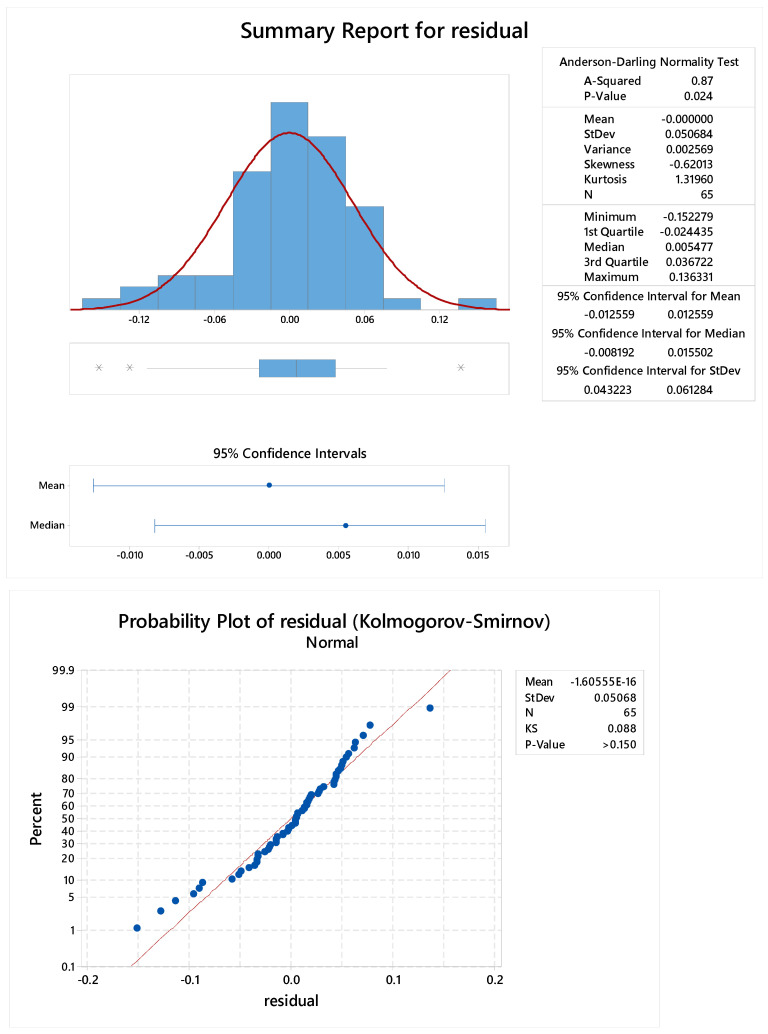
Residual distribution analysis for sample Linearity determination for quantification of *S. flexneri* 6 OAg by HPAEC-PAD (data from six analysis sessions together); * potential outliers.

**Figure A17:**
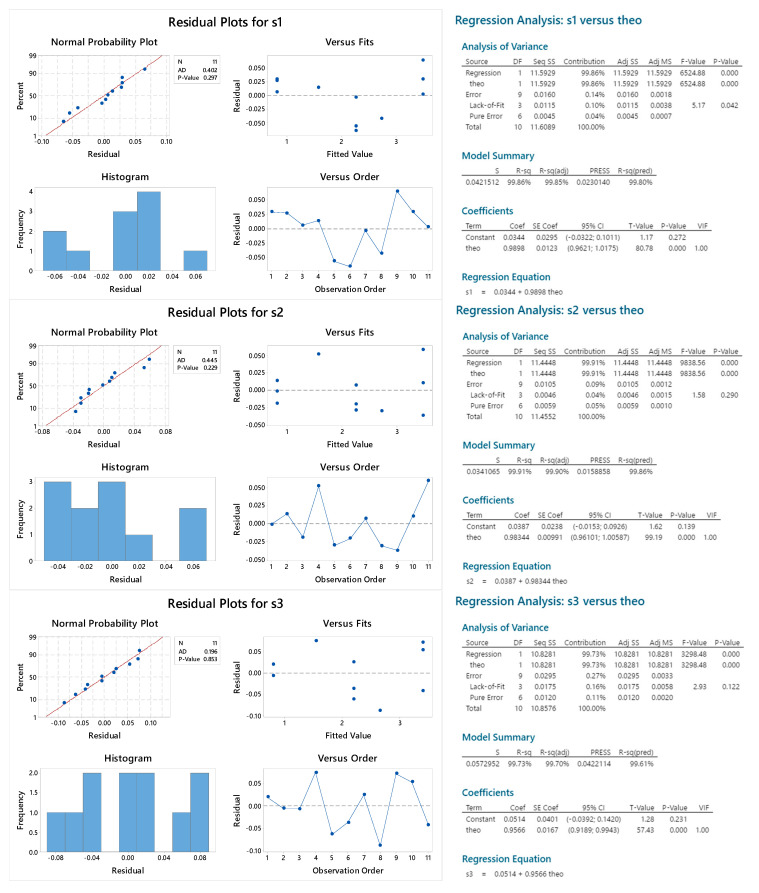
Linear regression analysis for sample Linearity determination for quantification of *S. flexneri* 6 OAg by HPAEC-PAD (data from single analysis session #1–3).

**Figure A18:**
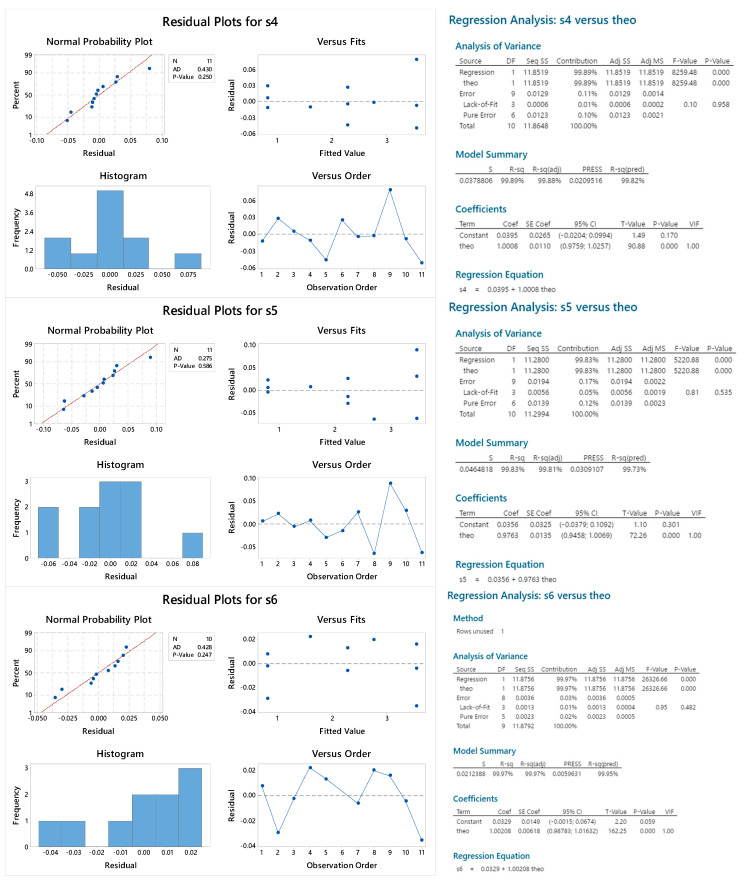
Linear regression analysis for sample Linearity determination for quantification of *S. flexneri* 6 OAg by HPAEC-PAD (data from single analysis session #4–6).

Reproducibility was calculated using a variance component analysis (REML, Figure A12) on data generated in six analysis sessions respectively at 1.00, 2.89 and 4.50 μg/mL concentration levels.

Sample linearity was determined on all data generated in the six analysis sessions, with an analysis of regression plotting the values measured against the theoretical concentrations calculated by sample dilution (six regressions were performed with data of single analysis session and one regression using data of all the analysis sessions). The true concentration of the more concentrated sample was assigned from the average of all its measurements for assessing sample linearity and reproducibility (18 values).

#### 4.3.8. Accuracy (Spike Recovery)

The spike recovery was evaluated on a glycoconjugate of *S. flexneri* 6 OAg conjugated to CRM_197_ carrier protein [17]. In four different analysis sessions the conjugate sample has been analyzed diluted to 1 μg/mL, without spiking as a reference and respectively spiked with 1, 2 and 3 μg/mL of *S. flexneri* 6 OAg standard (Table A4). Sample preparation order and chromatographic run order were randomized.

**Figure A19:**
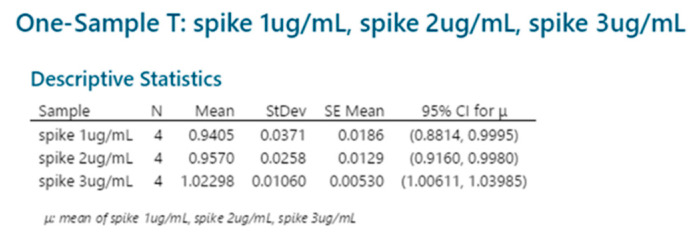
Spike recovery for HPAEC-PAD, confidence interval.

Confidence interval of the spike recovery values were calculated among the different analysis sessions (Figure A19).

### 4.4. Statistical Analysis

Statistical analyses were performed on Minitab v. 18.1.0 (Minitab Inc., State College, PA, USA) except for DoE that were planned and analyzed using Design Expert v. 10.0.3.1 (Stat-Ease, Minneapolis, MN, USA).

## Figures and Tables

**Table 1:** Variance component analysis for reproducibility of Dische colorimetric method.

Source	0.41 µg/mL		2.05 µg/mL		4.10 µg/mL	
	CV	*p* Value	CV	*p* Value	CV	*p* Value
Session	15.3%	0.093	0	-	0%	-
Repeatability	11.6%	0.009	7.7%	0.002	6.7%	0.002
Intermediate precision	19.2%		7.7%		6.7%	

**Table 2:** MS peak table assignment of *S. flexneri* 6 OAg hydrolyzed with TFA 2 M at 100 °C for 2 h.

	*m*/*z*	Theo. Mass	Delta (ppm)	Composition	Note
GalN	180.0866	180.0866	0	C_6_H_14_NO_5_	[MH]^+^
	162.0760	162.0761	−0.6	C_6_H_12_NO_4_	[MH-H_2_O]^+^
GalA-GalN	356.1181	356.1187	−1.7	C_12_H_22_NO_11_	[MH]^+^
	338.1077	338.1082	−1.5	C_12_H_20_NO_10_	[MH-H_2_O]^+^

**Table 3:** Variance component analysis for reproducibility of HPAEC-PAD method.

Source	1 μg/mL		2.89 μg/mL		4.5 μg/mL	
	CV	*p* Value	CV	*p* Value	CV	*p* Value
Session	0.9%	0.287	1.6%	0.114	1.0%	0.213
Repeatability	2.0%	0.007	1.4%	0.010	1.6%	0.007
Intermediate precision	2.3%		2.2%		1.8%	

**Table 4:** Determination of accuracy for the HPAEC-PAD method: spike recovery values at different spike concentrations.

	Spike		
	1 μg/mL	2 μg/mL	3 μg/mL
Recovery value (95% CI)	88.1–100%	91.6–99.8%	101–104%

## Data Availability

Not applicable.

## Appendix A

**Table A1:** Sample preparation and DoE runs for optimization of Dische colorimetric method.

Run	Type	Sample	Water	Cold H_2_SO_4_	Final H_2_SO_4_/Water	Cysteine 1 M	Warming Time
		μL	μL	μL	Ratio	μL	min
1	Factorial	200	205	1095	2.70	32.0	5.0
2	Factorial	200	205	1095	2.70	32.0	15.0
3	Axial	200	300	1000	2.00	36.8	10.0
4	Axial	200	300	1000	2.00	13.2	10.0
5	Factorial	200	452	848	1.30	18.0	5.0
6	Center	200	300	1000	2.00	25.0	10.0
7	Factorial	200	452	848	1.30	32.0	5.0
8	Center	200	300	1000	2.00	25.0	10.0
9	Axial	200	159	1141	3.18	25.0	10.0
10	Factorial	200	205	1095	2.70	18.0	15.0
11	Center	200	300	1000	2.00	25.0	10.0
12	Axial	200	300	1000	2.00	25.0	18.4
13	Axial	200	300	1000	2.00	25.0	1.6
14 ^1^	Factorial	200	205	1095	2.70	18.0	5.0
15	Factorial	200	452	848	1.30	18.0	15.0
16	Center	200	300	1000	2.00	25.0	10.0
17	Center	200	300	1000	2.00	25.0	10.0
18	Factorial	200	452	848	1.30	32.0	15.0
19	Axial	200	623	677	0.82	25.0	10.0
20	Center	200	300	1000	2.00	25.0	10.0

^1^ Data for fucose experiment run was not considered due to an error in the sample treatment.

**Table A2:** Sample preparation and DoE runs for HPAEC-PAD method.

Group	Run	Temperature °C	TFA %	HCl M	Time Min
1	1	50	10	6	150
1	2	50	10	6	150
1	3	50	10	6	150
2	4	50	10	6	150
2	5	50	10	6	150
2	6	50	10	6	150
3	7	50	10	6	150
3	8	50	10	6	150
3	9	50	10	6	150
4	10	60	11.5	4	240
4	11	60	20	8	240
4	12	60	0	8	160.8
4	13	60	0	4	60
4	14	60	20	6.2	60
5	15	50	10	6	150
5	16	50	10	6	150
5	17	50	10	6	150
6	18	50	10	6	150
6	19	50	10	6	150
6	20	50	10	6	150
7	21	40	20	8	60
7	22	40	10	6	150
7	23	40	0	8	240
7	24	40	20	4	240
7	25	40	0	4	60
8	26	60	8.5	8	240
8	27	60	0	4	240
8	28	60	20	4	159
8	29	60	12.9	8	60
8	30	60	0	5.92	60
9	31	40	0	8	60
9	32	40	0	4	240
9	33	40	20	4	60
9	34	40	20	8	240
9	35	40	10	6	150
10	36	60	9	4	60
10	37	60	20	4	60
10	38	60	0	6.3	240
10	39	60	0	8	60
10	40	60	20	5.68	240
10	41	60	20	8	141.9
11	42	50	10	6	150
11	43	50	10	6	150
11	44	50	10	6	150

**Table A3:** Statistical results for regression analysis (HPAEC-PAD method, sample linearity) on data from each analysis session.

	Session 1	Session 2	Session 3	Session 4	Session 5	Session 6
lack of fit	0.042	0.290	0.122	0.958	0.535	0.482
residual normality (*p*, AD)	0.297	0.229	0.853	0.250	0.586	0.247
linear coefficient (95% CI)	0.96–1.02	0.96–1.01	0.92–0.99	0.98–1.03	0.95–1.01	0.99–1.02
Intercept (95% CI)	−0.03–0.10	−0.02–0.09	−0.04–0.14	−0.02–0.10	−0.04–0.11	−0.002–1.02

**Table A4:** HPAEC-PAD recovery values per sessions.

	Spike		
μg/mL	1 μg/mL	2 μg/mL	3 μg/mL
Session 1	91%	92%	101%
Session 2	91%	97%	102%
Session 3	98%	95%	103%
Session 4	96%	98%	103%

**Table A5:** Sample replicate scheme used to generate data for Sample Linearity and Reproducibility.

Concentration	Session 1	Session 2	Session 3	Session 4	Session 5	Session 6
Level 1	3 replicates	3 replicates	3 replicates	3 replicates	3 replicates	3 replicates
Level 2	1 replicate	1 replicate	1 replicate	1 replicate	1 replicate	1 replicate
Level 3	3 replicates	3 replicates	3 replicates	3 replicates	3 replicates	3 replicates
Level 4	1 replicate	1 replicate	1 replicate	1 replicate	1 replicate	1 replicate
Level 5	3 replicates	3 replicates	3 replicates	3 replicates	3 replicates	3 replicates
